# Influence of diabetes mellitus duration on the efficacy of ischemic preconditioning in a Zucker diabetic fatty rat model

**DOI:** 10.1371/journal.pone.0192981

**Published:** 2018-02-23

**Authors:** Marie Vognstoft Hjortbak, Johanne Hjort, Jonas Agerlund Povlsen, Rebekka Vibjerg Jensen, Nicolaj Brejnholdt Støttrup, Mia R. Laursen, Nichlas Riise Jespersen, Bo Løfgren, Hans Erik Bøtker

**Affiliations:** 1 Department of Cardiology, Aarhus University Hospital, Skejby, Palle Juul-Jensens Blvd. Aarhus N, Denmark; 2 Department of Forensic Medicine, Aarhus University Hospital, Skejby, Palle Juul-Jensens Blvd. Aarhus N, Denmark; University of PECS Medical School, HUNGARY

## Abstract

Augmented mortality and morbidity following an acute myocardial infarction in patients with diabetes mellitus Type 2 (T2DM) may be caused by increased sensitivity to ischemia reperfusion (IR) injury or altered activation of endogenous cardioprotective pathways modified by T2DM *per se* or ischemic preconditioning (IPC). We aimed to investigate, whether the duration of T2DM influences sensitivity against IR injury and the efficacy of IPC, and how myocardial glucose oxidation rate was involved. Male Zucker diabetic fatty rats (homozygote (fa/fa)) at ages 6-(prediabetic), 12- (onset diabetes) and 24-weeks of age (late diabetes) and their age-matched non-diabetic controls (heterozygote (fa/+) were subjected to IR injury in the Langendorff model and randomised to IPC stimulus or control. T2DM rats were endogenously protected at onset of diabetes, as infarct size was lower in 12-weeks T2DM animals than in 6- (35±2% vs 53±4%; P = 0.006) and 24-weeks animals (35±2% vs 72±4%; P<0.0001). IPC reduced infarct size in all groups irrespective of the presence of T2DM and its duration (32±3%; 20±2%; 36±4% respectively; (ANOVA P<0.0001). Compared to prediabetic rats, myocardial glucose oxidation rates were reduced during stabilisation and early reperfusion at onset of T2DM, but these animals retained the ability to increase oxidation rate in late reperfusion. Late diabetic rats had low glucose oxidation rates throughout stabilisation and reperfusion. Despite inherent differences in sensitivity to IR injury, the cardioprotective effect of IPC was preserved in our animal model of pre-, early and late stage T2DM and associated with adaptations to myocardial glucose oxidation capacity.

## Introduction

Ischemic heart disease (IHD) is the leading cause of death worldwide. A serious manifestation of IHD is acute myocardial infarction (AMI). Patients with T2DM not only have an increased prevalence of IHD, but also increased mortality and morbidity following AMI, mainly due to an increased incidence of post infarction heart failure[1]. The underlying mechanisms are multifactorial and may involve accumulation of risk factors[2] and more complex and extensive coronary artery disease than in non-diabetic patients[3]. However, outcome may also be influenced by increased sensitivity to ischemia reperfusion (IR) injury or altered activation of endogenous cardioprotective pathways by diabetes mellitus type 2 (T2DM) *per se* or by ischemic preconditioning (IPC). IPC can be applied as an exogenous intervention by one or more cycles of brief ischemia that renders the myocardium less susceptible to a subsequent prolonged ischemia and reperfusion (IR) event[4]. IPC also appears as an inherent activator of endogenous protective mechanism as seen in patients with preinfarction angina, who may suffer minor myocardial infarcts[5–7] and better outcome than those without following an AMI[8,9].

The efficacy of IPC is altered in the presence of comorbidities such as hyperlipidemia, hypertension, obesity and ageing[10]. Particularly in T2DM, the efficacy of IPC varies in experimental models[11], whereas the variability seems less manifest in clinical studies[12,13]. Several inherent features of T2DM have been proposed to explain the reduced sensitivity of IPC in experimental models of T2DM[11]. The mechanisms underlying IPC include recruitment of endogenous protective pathways that ultimately converge on the mitochondrial permeability transition pore and inhibit its opening. Preservation of mitochondrial and cellular integrity limits infarct size[14,15]. Mitochondrial dysfunction is considered inherent to the pathophysiology of T2DM[16], and may be one of the mechanisms that modify metabolic flexibility[17], which seems to be a prerequisite for modification of metabolism to elicit cardioprotection[18]. We have found that sensitivity to ischemia varies with the duration of T2DM, such that sensitivity appears to be similar or even decreased compared to non-diabetic (non-DM) individuals at onset of T2DM and seriously increased in late stage unregulated T2DM[19]. We hypothesized that diabetes duration modifies the efficacy of IPC depending on the variable degree of endogenous cardioprotection related to duration of T2DM and changes in myocardial glucose oxidation. To investigate whether the efficacy of exogenously applied IPC was influenced by the duration of T2DM, we studied infarct size, hemodynamics, glucose oxidation and whether the efficacy of IPC was reflected in changes in interstitial succinate concentrations, a key mediator of IR injury[20], in a Zucker diabetic fatty rat model of T2DM. We found that at onset of diabetes the animals were endogenous protected against IR-injury compared to pre-diabetes and late-diabetes. The effect of IPC was not influenced by this variation in endogenous cardioprotection, but provided cardioprotection irrespective of the duration of T2DM.

## Materials and methods

### Ethics statement

Animals were handled in accordance with national and institutional guidelines for animal research. The Danish Animal Experiments Inspectorate approved the experimental work (license no. 2011/561-2010-C2).

### Animals

Male Zucker diabetic fatty (ZDF) rats (homozygote (fa/fa)) where used at ages 6-weeks (prediabetic), 12-weeks (onset diabetes) and 24-weeks (late diabetes) and their age-matched non-diabetic controls (heterozygote (fa/+)) (Charles River Laboratories, Kislegg, Germany). All rats were housed in pairs under controlled conditions with 12:12 h light-dark cycles and kept on an ad libitum diet of Purina 5008 as recommended by the supplier. The rats were subjected to 12–14 hours of fasting prior to experimental procedures and did not at any point receive anti-diabetic treatment.

### Experimental protocols

**Fig 1:**
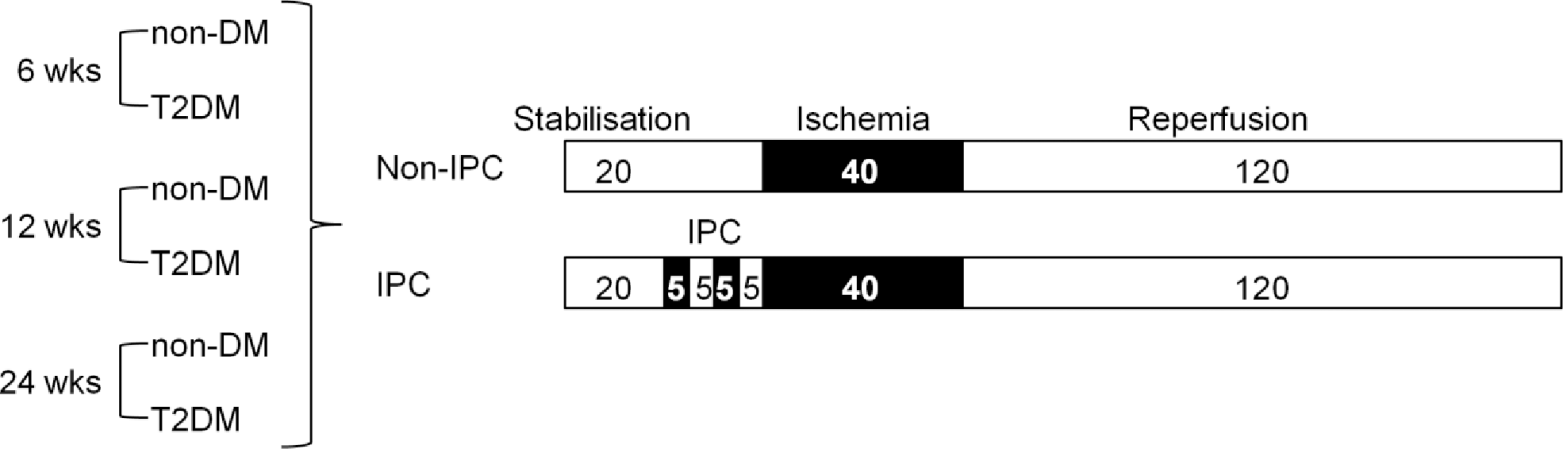
Study design. Overview of experimental groups and protocols.

Prior to surgery hearts were randomised to an IPC or no IPC group. Each group consisted of 8–11 animals. Hearts were allowed to stabilise for 40 min prior to 40 minutes of no-flow global ischemia and 120 minutes of reperfusion. In the intervention group, IPC was induced by two cycles of 5 min ischemia + 5 min reperfusion prior to index ischemia (Fig 1).

Hearts that did not achieve a left ventricular developed pressure of minimum 120 mmHg during the first 20 min of stabilisation and/or suffered intractable VF/VT during reperfusion were excluded from analyses.

### Analysis of blood glucose and plasma metabolites

To validate the disease model preanesthetic tail vein blood was drawn for measurement of glucose (One Touch® Ultra Blood Glucose, Lifescan Inc., CA, USA) and insulin levels (DRG Instruments, Marburg, Germany) at the beginning of each test day and no later than an hour prior to surgery. Plasma total cholesterol and triglycerides were measured in blood samples drawn from the abdominal aorta during surgery. Preparation for insulin and lipid profile measurements where done as previously described[19].

### Isolated heart model

All rats were anaesthetized by a subcutaneous injection of a mixture of Dormicum® (midazolam, 0,5 mg/kg, Matrix Pharmaceuticals, Herlev, DK), Hypnorm® (fentanylcitrate, 0,158 mg/kg and fluanisone 0,5 mg/kg, Vetapharma Ltd., Leeds, UK), and sterile water (dosage 0,2 mL mixture/100 g body mass). The rat was connected to a ventilator (Ugo Basile 7025 rodent ventilator, Comerio, Italy) and through a thoracotomy the heart was dissected free from surrounding structures and cannulated in situ according to standard procedure in our laboratory. Hearts were perfused with 37°C Krebs-Henseleit (KH) buffer (11.1 mM glucose) and supplied at a constant pressure of 80 mmHg.

A balloon catheter connected to a pressure transducer was inserted into the left ventricular cavity to assess left ventricular function. Diastolic pressure was pre-set to 8–10 mmHg during stabilisation. Coronary flow was measured using an inline flowmeter (Hugo Sacs Electronic, March-Hugstetten, DE), and hemodynamic data was acquired and analysed using Notocord®-hem software (Notocord, Croissy- Sur-Seine, France).

### Infarct size measurement

After completion of protocol hearts were frozen and sliced before undergoing staining with 2,3,5-triphenyltetrazoliumchloride (TTC). After staining, the hearts were stored in a 4% formaldehyde solution (Lillies Solution, VWR Bie & Berntsen, Herlev, Denmark) for approximately 24 hours. IS was analysed by manual delineation using image analysis software (ImageJ, NIH).

### Glucose oxidation

Rates of glucose oxidation were measured using a strategically tritium labelled glucose isotope (D-[6- 3H]-glucose).[21] A buffer volume of 1500 mL (75μL D-[6-3H]-glucose/1500 mL KH buffer) was recirculated. Pre-experimental buffer samples were obtained to calculate baseline specific activity (SA) per μmol glucose. Glucose oxidation was quantified by ^3^H2O production from oxidation of D-[6-3H]-glucose in the citric acid cycle. This was done using scintillation-techniques, as described comprehensively elsewhere.^13^ Results were weighted against the mass of each individual heart (wet weight). Samples from both the inline flow tube (“arterial” (A)) and the coronary effluent (“venous” (V)) were recovered during the first 5 minutes of reperfusion to visualise the dynamic oxidation during early reperfusion. These results were corrected for heart weight and coronary flow.

### Microdialysate

A microdialysis catheter (membrane length 4 mm, 6 kDa cutoff; AgnTho’s AB, SE) was implanted into the left ventricular free wall during instrumentation and continuously perfused (1 μL/min) with deoxygenated KH buffer. Samples were collected every 10 min during the experimental protocol and stored at -80°C until further analysis. Concentrations of lactate and succinate were determined by liquid chromatography/electrospray-tandem mass spectrometry as described comprehensively elsewhere[22]. The final interstitial concentrations were calculated by correction for relative recovery rate (lactate 37%, succinate 26%).

### Statistics and calculation

Data are presented as mean ± SEM, unless otherwise indicated. Data were compared using ANOVA with a post hoc test when appropriate (Turkey’s multiple comparisons test) and ANOVA with repeated measurements (or equivalent non-parametric test). All statistical calculations were performed using GraphPad Prism (GraphPad Software, CA, USA). P<0.05 was considered significant. The required sample size was estimated from previously published work using the isolated heart model[19].

## Results

### Animal characteristics

Animal characteristics are shown in Table 1. In both T2DM rats and their lean age-matched non-DM rats bodyweight increased with age. T2DM rats had higher bodyweight than their controls at all ages, but heart/bodyweight ratio was only significantly increased at 6- and 12-weeks.

**Table 1:** Animal characteristics.

	6 Weeks		12 Weeks		24 Weeks	
	Non-DM	ZDF	Non-DM	ZDF	Non-DM	ZDF
	*(n = 19)*	*(n = 18)*	*(n = 18)*	*(n = 20)*	*(n = 19)*	*(n = 18)*
Bodyweight (g)	151±24	186±25 **	309±13	358±21 **	434±20	405±30 **
Heartweight (mg)	697±174	726±163	1095±147	1091±73	1425±306	1312±344
HW/ BW. ratio^†^	4.3±0.4	3.5±0.4 **	3.5±0.3	3.0±0.2 **	3.2±0.3	3.1±0.3
B-glucose (mmol/L)	4.8±0.5	6.5±1.3	4.9±0.4	17.3±6.3 **	5.3±0.3	19.9±4.5 **
P-total cholesterol (mmol/L)	2.0±0.6	2.4±0.9	1.7±0.3	3.7±0.7 **	2.4±1.0	6.8±1.4 **
P-triglyceride (mmol/L)	0.5±0.2	1.9±0.8 *	0.5±0.07	6.2±2.0 **	0.7±0.2	7.5±3.0 **
P-Insulin (pmol/L)	16.0±16	356±145 **	22±21	130±146 *	32±34	55±55

Mean ± SD.

*p< 0.05 compared to age-matched controls.

**p<0.005 compared to age-matched controls

^†^ HW/BW ratio: Heart weight / body weight

As expected, we observed no difference in the blood-glucose levels in the prediabetic 6-week-old ZDF rats compared to lean non-DM age-matched controls, but onset diabetic, 12-week-old, and late diabetic, 24-week-old, T2DM rats had higher blood-glucose levels than non-DM lean controls. Plasma-cholesterol concentrations increased with age in T2DM rats and were higher at all ages than their non-DM controls.

Due to technical challenges with the microdialysis analysis and glucose oxidation measurements, we experienced a few missing values. Baseline characteristics for these sub-groups did not differ from the entire cohort, indicating that we did not introduce significant selection bias (S1 Table).

### Infarct size

**Fig 2:**
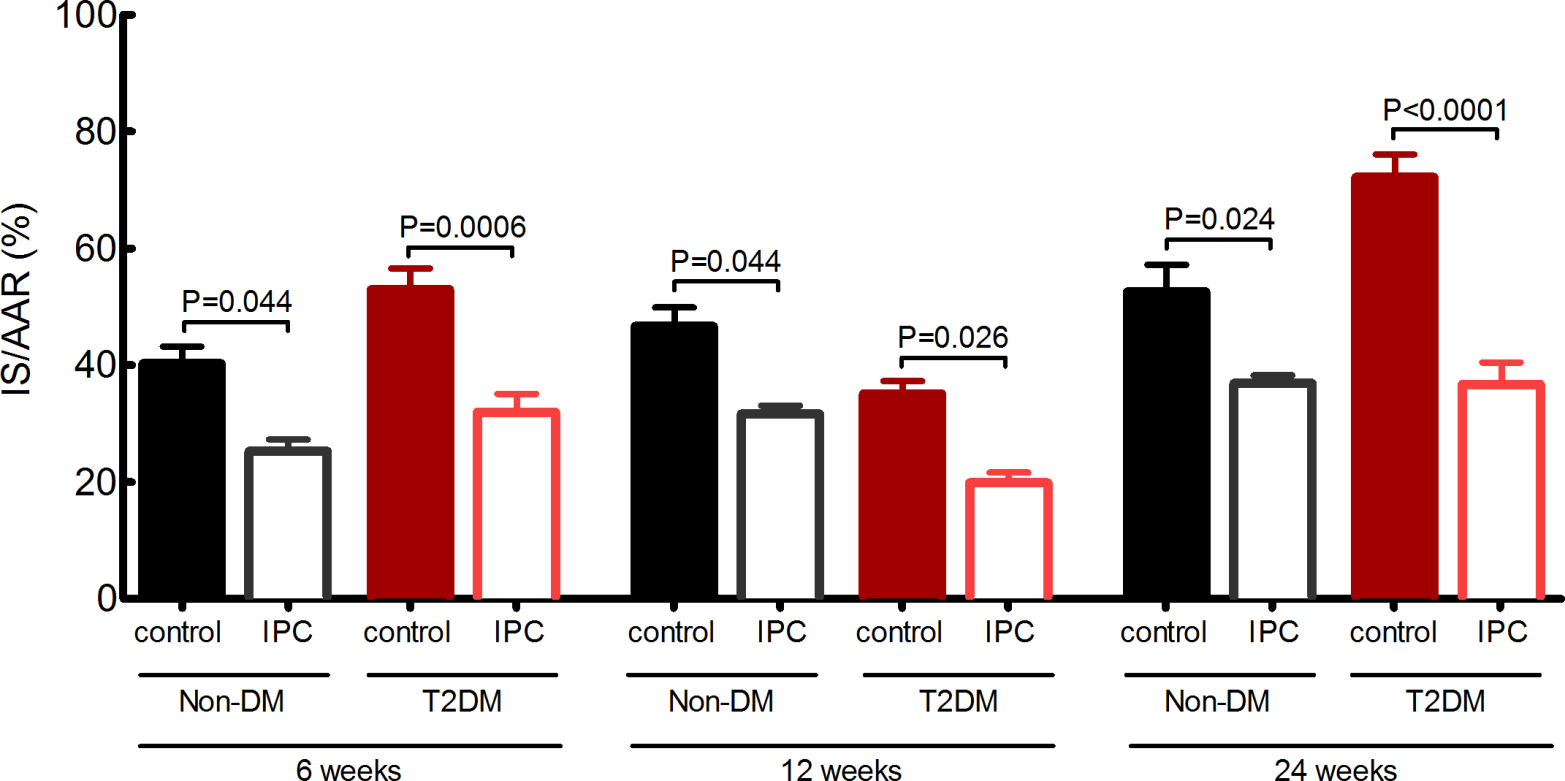
Infarct size. Infarct size as a ratio of area at risk in 6-, 12- and, 24-week-old non-DM rats (heterozygote (fa/+)) and DM ZDF rats (homozygote (fa/fa)) treated with control or IPC protocol. P-values are post hoc analysis between groups of interest. Data are mean ± SEM (n = 8–11).

In non-DM animals infarct size increased with age, but the increment was of borderline statistical significance (40±3%; 47±3%; 53±5%, 6-, 12-, 24-weeks respectively; ANOVA P = 0.07)(Fig 2). Diabetes modified the susceptibility to IR injury (53±4%; 35±2%; 72±4%, 6-, 12-, 24-weeks respectively; ANOVA P<0.0001), and the pattern differed from non-DM animals (ANOVA P<0.0001). At ages 6- and 12-weeks, we found no difference in IS between non-DM and T2DM rats (40±3% vs 53±4%; P = 0.10 and 47±3% vs 35±2%; P = 0.17 respectively), while infarct size was significantly higher in late diabetic rats than in age-matched lean non-DM controls (53±5 vs 72±4%; P = 0.004). At onset of diabetes at 12-weeks, T2DM rats were endogenously protected, as infarct size was lower than in T2DM animals at 6- (35±2% vs 52±4%; P = 0.006) and 24-weeks (35±2 vs 72±4%; P<0.0001).

IPC reduced infarcts size in all groups irrespective of the presence of T2DM and its duration (non-DM: 40±3% vs 25±2%; 47±3% vs 32±1%; 53±5% vs 37±1%; T2DM: 53±4% vs 32±3%; 35±2% vs 20±2%; 72±4% vs 36 ±4%; 6-, 12-, 24-weeks respectively; ANOVA P<0.0001)(Fig 2).

### Hemodynamics

#### Preischemically

For non-DM animals, rate pressure product (RPP) during stabilisation was similar at 6- and 12-weeks, and decreased in 24-week animals (ANOVA P = 0.0009) (Table 2). Similarly, RPP was not different for 6- and 12-week T2DM animals, but decreased at 24-weeks (ANOVA P = 0.04). The decrease in RPP at 24-weeks was caused by a decrease in heart rate (HR), as HR was lower in 24-week both non-DM and T2DM animals (ANOVA P = 0.04 and P = 0.03, respectively), while left ventricular pressure (LVDP) was unchanged.

**Table 2:** Hemodynamic parameters preischemically and during postischemic-reperfusion.

		n	Baseline	Reperfusion						
				2 min	5 min	10 min	20 min	30 min	60 min	120 min
**LVDP (mmHg)**										
6 Weeks	non-DM control	11	137 ± 3	5 ± 1	6 ± 1	7 ± 1	32 ± 5	59 ± 3	60 ± 2	48 ± 2
	non-DM IPC	8	132 ± 4	11 ± 3	27 ± 6	49 ± 8	83 ± 6	79 ± 5	70 ± 5	57 ± 5
	T2DM control	10	155 ± 6	7 ± 2	7 ± 2	7 ± 2	12 ± 4	26 ± 6	33 ± 3	25 ± 3
	T2DM IPC	8	156 ± 7	7 ± 2	9 ± 2	10 ± 2	37 ± 4	50 ± 4	48 ± 2	37 ± 2
12 Weeks	non-DM control	10	160 ± 4	5 ± 1	27 ± 3	20 ± 2	46 ± 6	61 ± 7	66 ± 6	55 ± 4
	non-DM IPC	9	151 ± 7	13 ± 2	34 ± 5	39 ± 5	52 ± 6	64 ± 4	66 ± 4	51 ± 3
	T2DM control	11	167 ± 6	9 ± 2	23 ± 3	28 ± 5	42 ± 6	61 ± 7	68 ± 3	54 ± 5
	T2DM IPC	8	174 ± 7	7 ± 1	21 ± 5	24 ± 3	55 ± 6	74 ± 10	73 ± 4	70 ± 7
24 Weeks	non-DM control	10	132 ± 8	5 ± 1	15 ± 3	12 ± 2	21 ± 8	30 ± 8	47 ± 4	40 ± 3
	non-DM IPC	9	162 ± 4	13 ± 2	20 ± 5	12 ± 3	22 ± 6	13 ± 2	54 ± 4	53 ± 4
	T2DM control	9	157 ± 7	11 ± 2	13 ± 3	16 ± 2	16 ± 4	22 ± 4	32 ± 6	22 ± 4
	T2DM IPC	8	146 ± 1	9 ± 3	25 ± 7	40 ± 7	58 ± 8	68 ± 8	55 ± 8	56 ± 5
**RPP (0.01xmmHgxmin-1)**										
6 Weeks	non-DM control	11	373 ± 10	9 ± 1	12 ± 2	16 ± 3	71 ± 15	153 ± 12	166 ± 10	126 ± 7
	non-DM IPC	8	298 ± 27	29 ± 10	81 ± 19	136 ± 21	213 ± 17	211 ± 22	194 ± 17	158 ± 17
	T2DM control	10	333 ± 29	14 ± 4	13 ± 3	11 ± 2	21 ± 7	53 ± 12	76 ± 6	54 ± 9
	T2DM IPC	8	347 ± 23	17 ± 5	23 ± 6	25 ± 5	92 ± 10	119 ± 12	126 ± 10	106 ± 5
12 Weeks	non-DM control	10	473 ± 63	12 ± 2	55 ± 8	45 ± 7	107 ± 12	147 ± 16	160 ± 13	123 ± 11
	non-DM IPC	9	308 ± 28	31 ± 5	79 ± 14	87 ± 16	115 ± 13	140 ± 12	174 ± 18	113 ± 13
	T2DM control	11	272 ± 23	20 ± 3	55 ± 12	54 ± 13	66 ± 9	93 ± 13	115 ± 10	94 ± 12
	T2DM IPC	9	203 ± 14	15 ± 2	39 ± 10	47 ± 6	74 ± 10	129 ± 19	151 ± 17	84 ± 12
24 Weeks	non-DM control	10	246 ± 18	11 ± 1	39 ± 9	32 ± 5	34 ± 11	59 ± 17	79 ± 10	67 ± 10
	non-DM IPC	9	273 ± 27	34 ± 6	56 ± 13	35 ± 13	59 ± 16	34 ± 7	149 ± 15	124 ± 20
	T2DM control	9	231 ± 25	22 ± 3	16 ± 4	16 ± 4	15 ± 4	22 ± 5	32 ± 7	34 ± 7
	T2DM IPC	8	184 ± 25	16 ± 4	31 ± 7	51 ± 13	67 ± 14	82 ± 17	96 ± 23	123 ± 14
**HR (bpm)**										
6 Weeks	non-DM control	11	273 ± 4	198 ± 19	216 ± 11	230 ± 10	213 ± 16	255 ± 10	272 ± 10	261 ± 8
	non-DM IPC	8	225 ± 19	244 ± 10	292 ± 13	287 ± 13	258 ± 15	267 ± 21	278 ± 16	279 ± 12
	T2DM control	10	217 ± 18	194 ± 17	225 ± 17	195 ± 23	201 ± 19	197 ± 9	231 ± 11	217 ± 10
	T2DM IPC	8	226 ± 19	236 ± 15	237 ± 17	249 ± 11	252 ± 11	238 ± 19	265 ± 19	288 ± 6
12 Weeks	non-DM control	9	292 ± 34	219 ± 20	205 ± 18	241 ± 31	246 ± 17	229 ± 8	231 ± 4	224 ± 7
	non-DM IPC	9	209 ± 20	230 ± 12	230 ± 27	235 ± 27	230 ± 22	221 ± 18	264 ± 21	229 ± 13
	T2DM control	11	166 ± 16	234 ± 16	236 ± 32	209 ± 26	170 ± 21	154 ± 12	170 ± 11	182 ± 25
	T2DM IPC	8	123 ± 12	237 ± 14	231 ± 41	234 ± 35	147 ± 9	206 ± 30	219 ± 20	128 ± 28
24 weeks	non-DM control	10	216 ± 13	244 ± 11	263 ± 16	268 ± 19	229 ± 31	222 ± 17	195 ± 29	189 ± 25
	non-DM IPC	9	169 ± 16	270 ± 9	285 ± 22	269 ± 18	258 ± 34	237 ± 17	289 ± 7	291 ± 18
	T2DM control	9	150 ± 17	224 ± 18	137 ± 22	109 ± 17	138 ± 27	121 ± 25	128 ± 27	177 ± 32
	T2DM IPC	8	129 ± 16	205 ± 30	158 ± 35	119 ± 14	114 ± 14	117 ± 15	190 ± 36	232 ± 30

Preischemic hemodynamic variables in 6-, 12-, and 24-week-old non-DM rats (heterozygote (fa/+)) and DM ZDF rats (homozygote (fa/fa)) treated with control or IPC. LVDP: Left ventricular developed pressure, RPP: Rate-pressure-product, HR: Heart rate.

Data are mean ± SEM.

Compared to non-DM controls, T2DM animals at 12-weeks had lower RPP at stabilisation (ANOVA P = 0.002), which was caused by a decrease in HR (P = 0.04) and no difference in LVDP (Table 2).

#### Postischemically

In non-DM animals RPP during reperfusion was similar for 6- and 12-week animals, and decreased in 24-week animals (ANOVA P<0.0001)(Table 2), with corresponding decreases in both LVDP (ANOVA P = 0.0001). In T2DM animals, RPP was similar in 6- and 24-week animals, and increased in 12-week animals (ANOVA P<0.0001) in accordance with smaller infarct size. The increase in RPP in 12-week T2DM animals was mainly due to an increase in LVDP (ANOVA P<0.0001).

In 6-week animals, IPC increased RPP in both non-DM and T2DM animals (ANOVA P<0.0001 and P = 0.01, respectively). Similarly, RPP increased in 24-week non-DM and T2DM animals (ANOVA P = 0.005 and P<0.0001, respectively), whereas IPC did not change RPP at age 12-weeks (ANOVA P = 0.86 and P = 0.79, respectively) (Table 2).

### Exogenous glucose oxidation

#### Preischemically

**Fig 3:**
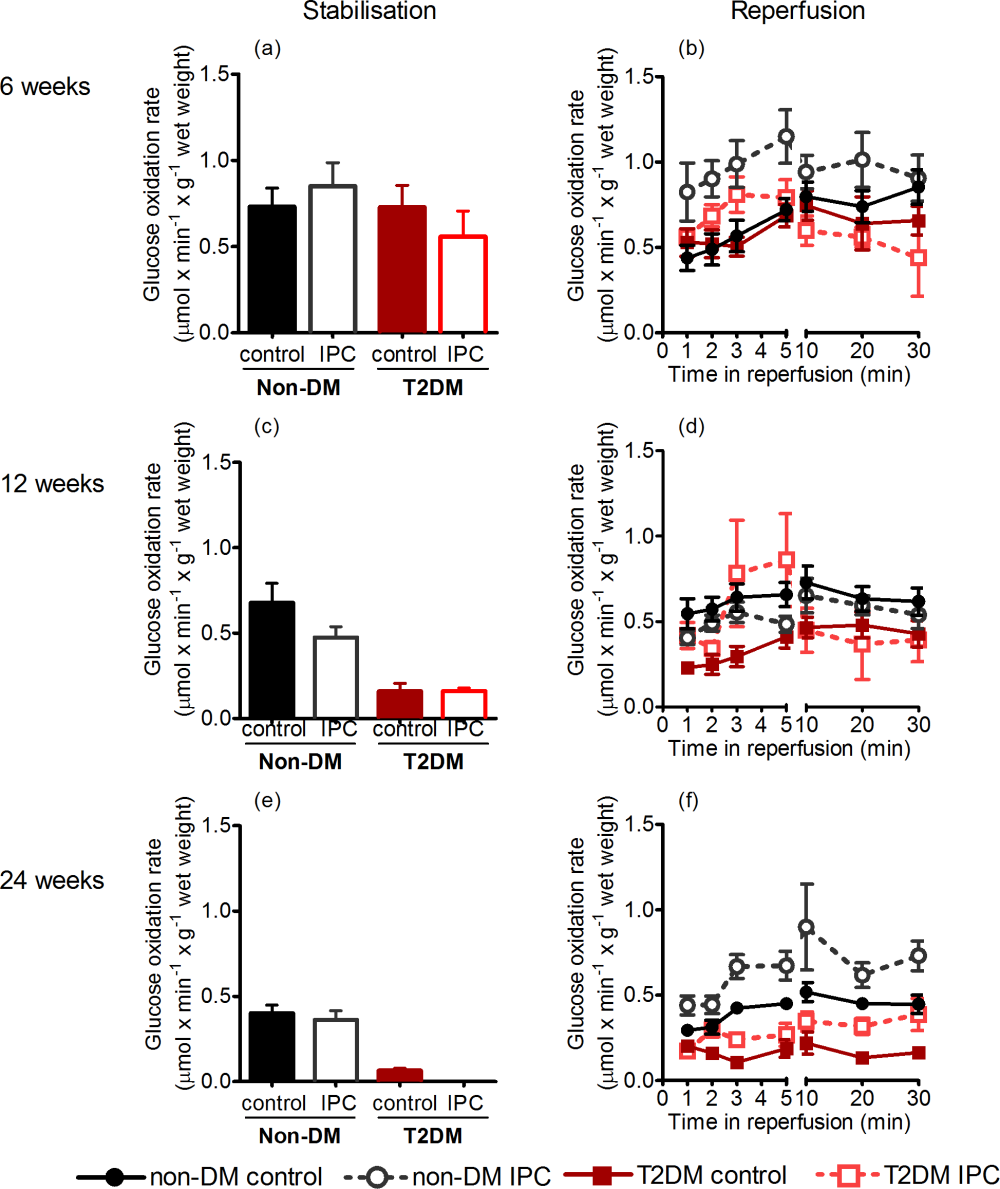
Glucose oxidation rates. Tracer estimated glucose oxidation rates preischemically and during stabilisation in 6- (a+b), 12- (c+d), and 2-week-old (e+f) non-DM rats (heterozygote (fa/+)) and DM ZDF rats (homozygote (fa/fa)) after control or IPC-treatment. Data are mean ± SEM (n = 7–10).

Exogenous myocardial glucose oxidation was similar in non-DM rats at 6 and 12 weeks (ANOVA P = 0.91), but reduced with age at 24-weeks (ANOVA P = 0.049) (Fig 3A, 3C and 3E). In T2DM rats, glucose oxidation was already reduced at 12-weeks (ANOVA P<0.0001), with a further reduction at 24-weeks (ANOVA P<0.0001). In 24-week T2DM rats, glucose oxidation rates were below detection level in the IPC group.

#### Postischemically

During reperfusion, we found no difference in glucose oxidation rates in non-DM rats at 6- and 12-weeks of age, while oxidation rate decreased at 24-weeks of age (ANOVA P = 0.007) (Fig 3).

Non-DM rats and T2DM rats had similar oxidation rates at 6 weeks of age (P = 0.97). At 12- and 24-weeks, T2DM rats had lower oxidation rates than non-DM rats (P = 0.009 and P = 0.0008, respectively).

For 12-week T2DM rats the decrease in oxidation rate was predominant during the first 2 minutes; subsequently the rates increased temporarily and did not differ from non-DM rats during the remaining 10–30 minutes of the reperfusion. At 24-weeks, T2DM rats had low oxidation rates throughout the first 30 minutes of reperfusion. IPC increased glucose oxidation rates during the first five minutes of reperfusion in both non-DM and T2DM rats at all ages, except in 12-week non-DM rats. In the subsequent 10–30 minutes period of reperfusion, glucose oxidation rates did not differ between the groups at 6- and 12-weeks of age, while oxidation rates remained higher with IPC in non-DM group but not statistically significantly in the T2DM group at 24-weeks (Fig 3).

### Interstitial metabolite concentration

**Fig 4:**
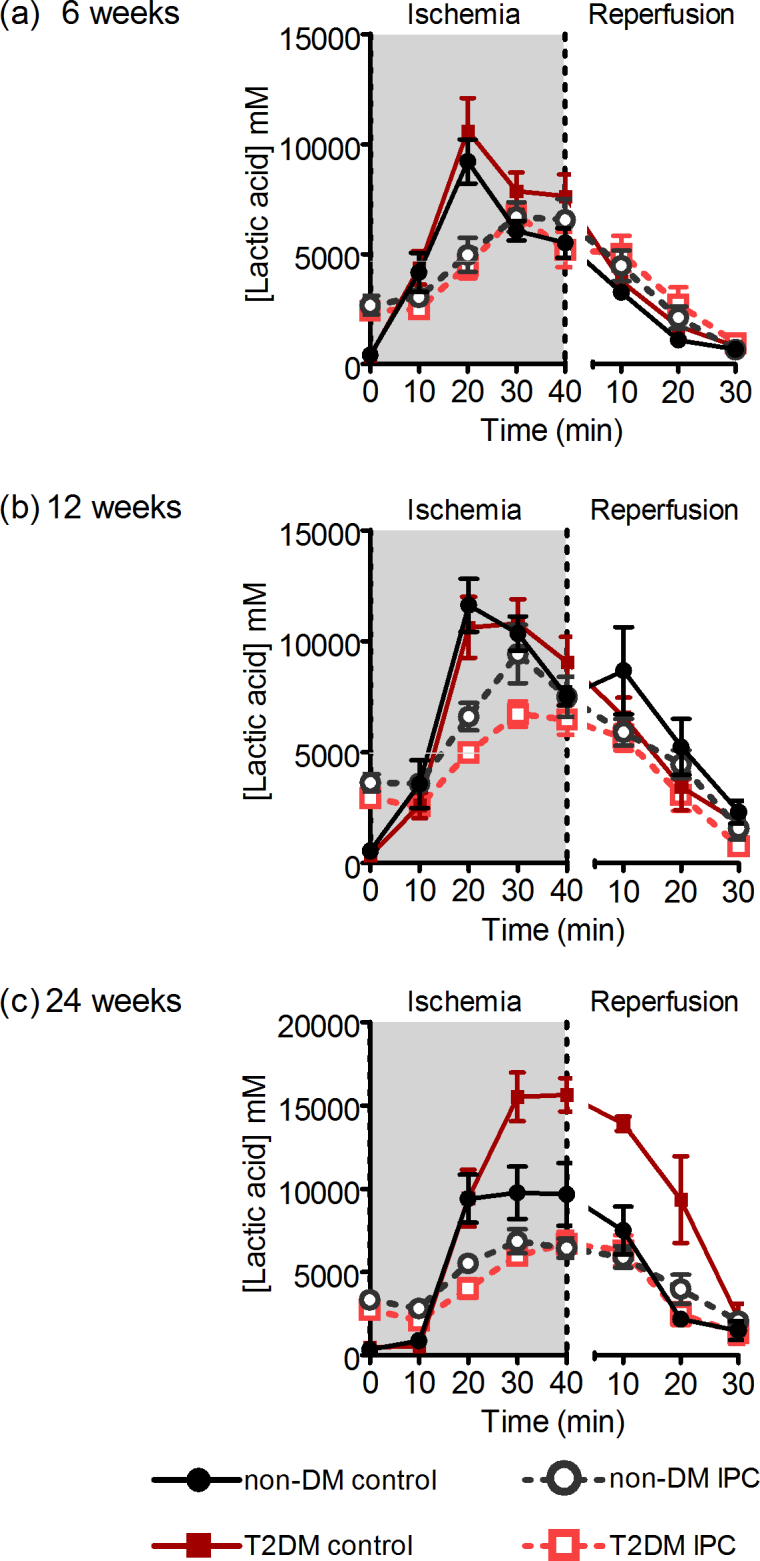
Interstitial lactate concentrations. Interstitial concentrations of lactate during ischemia and reperfusion in 6- (a), 12- (b), and 24-week-old (c) non-DM rats (heterozygote (fa/+)) and DM ZDF rats (homozygote (fa/fa)) after control or IPC-treatment. Data are mean ± SEM (n = 8–11).

Throughout ischemia, we found no difference in lactate concentration in non-DM rats irrespective of age (ANOVA P = 0.16). At ages 6- and 12-weeks, T2DM and non-DM rats had similar interstitial lactate concentrations (P = 0.18 and P = 0.99, respectively). At 24-weeks, the increment in interstitial lactate concentration was significantly higher in T2DM than in non-DM rats (P<0.05). IPC reduced the interstitial concentration of lactate at all ages (ANOVA P<0.0001) (Fig 4).

**Fig 5:**
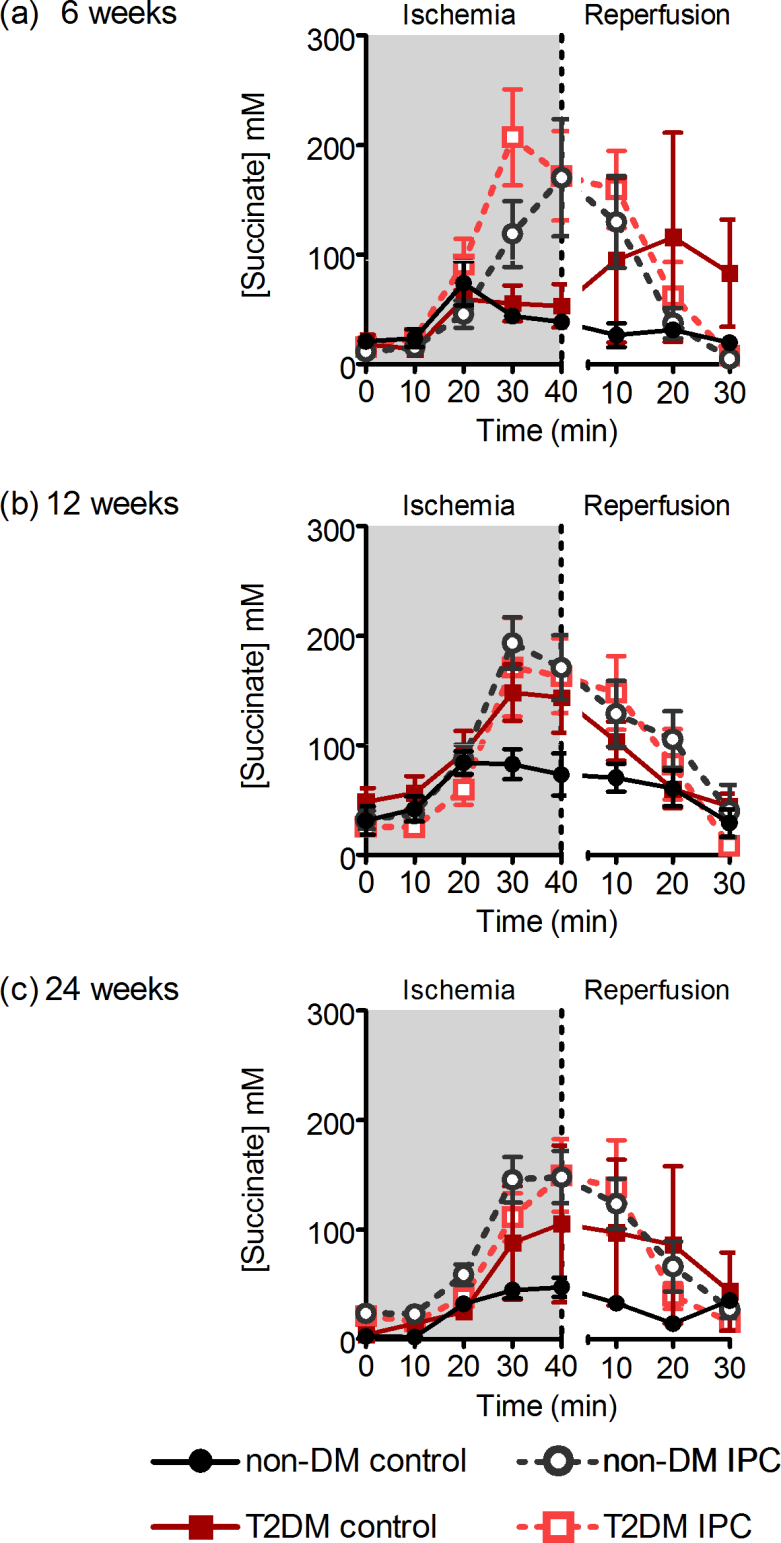
Interstitial succinate concentrations. Interstitial concentrations of succinate during ischemia and reperfusion in 6- (a), 12- (b), and 24-week-old (c) non-DM rats (heterozygote (fa/+)) and DM ZDF rats (homozygote (fa/fa)) after control or IPC-treatment. Data are mean ± SEM (n = 2–9).

During ischemia the change in succinate concentrations did not differ between T2DM and non-DM rats at 6-weeks (ANOVA P = 0.62), increased statistically borderline significantly at 12-weeks (ANOVA P = 0.06) and at 24-weeks (ANOVA P = 0.08). No differences were apparent during reperfusion. IPC significantly increased interstitial succinate concentrations in non-DM rats at all ages (ANOVA P = 0.03 at 6-weeks, P = 0.02 at 12-weeks and P<0.0001 at 24-weeks). In T2DM rats, IPC increased interstitial succinate concentrations at 6-weeks (ANOVA P = 0.01). Although IPC increased interstitial succinate concentrations additive to the increase of diabetes *per se*, the increment did not achieve statistical significance at 12-weeks (ANOVA P = 0.85) or 24-weeks (ANOVA P = 0.51) (Fig 5).

## Discussion

The novel finding of the present study is that IPC provides cardioprotection to both non-DM rats and T2DM rats at all ages and irrespective of the duration of T2DM. Thus, the underlying mechanisms of IPC are not blocked by the metabolic phenotype of T2DM notwithstanding decreased sensitivity against IR injury at onset or increased sensitivity at late stage diabetes.

Previous studies have demonstrated either no effect of conditioning[23] or that a more powerful stimulus is required to provide cardioprotection[24,25]. The effect of ischemic preconditioning in DM seems to vary in experimental settings. In animals with a short duration of diabetes, glucose as the only substrate and a no-flow IR protocol, DM was associated with less sensitivity to IR injury[10]. In animals with a prolonged duration of diabetes, a low-flow IR protocol with free fatty acids present in the perfusate demonstrated an increased sensitivity to IR injury[11]. These considerations also apply in our study. We conducted the study in an isolated heart model, with glucose as the sole substrate, which determines the substrate preference of the myocardial metabolism. Our model enabled us to evaluate associations between infarct size modifications and the adaptations in glucose metabolism. The ZDF rat model reflects a combination of several comorbidities that might interfere with sensitivity towards IR injury, but also conditioning strategies. The rats suffer not only from T2DM with high blood glucose and hyperinsulinemia, they also have elements of metabolic syndrome with obesity and elevated levels of cholesterol and triglycerides. Such comorbidities are also characteristic for patients with T2DM, which may help the translation of the results to clinical use. The animals develop T2DM very rapidly compared to humans. However, ages of 6-, 12-, and 24-weeks still reflect young animals, whereas the incidence of T2DM rises later in life in humans.

Because many studies have demonstrated impaired efficacy of IPC in diabetic animals, several underlying mechanisms have been proposed[11]. IPC modifies intracellular pathways that may be modulated by diabetes[26–29]. Dysfunctional mitochondria and generation of ROS is known to be a part of the increased sensitivity of diabetic myocardium to IR injury[16]. As the cardioprotective mechanisms of IPC converge towards the mitochondria, this may also be a reason for decreased effect of IPC in T2DM. The alterations in the myocardium of diabetes may vary between animal models and with disease severity, and give rise to conflicting data. A similar variation in several of these elements is likely in diabetic patients, and an optimisation of the IPC strategy may be needed to gain full effect of IPC in clinical practice.

In the present study, we demonstrated that IPC does not interact with variation of endogenous cardioprotection and provides cardioprotection irrespective of the duration of T2DM. In an earlier study, we found that IPC did not yield protection in a similar ZDF animal model as used in the present study, but in 16-week ZDF rats[23]. The reason for the lack of effect of IPC in our earlier study may not only be due to differences in age, but also in different ischemia exposure and the use of a less intense IPC stimulus.

When comparing non-DM and T2DM animals, we identified differences in glucose oxidation patterns that may be involved in the mechanisms underlying differences in sensitivity to IR injury between T2DM and non-DM animals. In the prediabetic state, glucose oxidation did not differ from age-matched lean controls, but at 12-weeks, i.e. at onset of diabetes, T2DM rats had decreased glucose oxidation rates during stabilisation and early reperfusion, but still had the ability to increase oxidation rate later in reperfusion. At age 24-weeks, the T2DM animals had very low rates of glucose oxidation during stabilisation, and the hearts did not show improvement during reperfusion. These findings may reflect that endogenous protection against IR injury at onset diabetes could be due to optimisation of metabolic substrate utilization with alterations in glucose metabolism, whereas this modulation of metabolism is impaired in late diabetes. The severe reduction in glucose oxidation rates in late stage T2DM rats suggests a link between increased sensitivity to IR injury and diminished metabolic flexibility in late stage T2DM. Such disarrays may be associated with dysfunction of the mitochondria. Similar to 24-week T2DM rats, non-DM rats also had reduced glucose oxidation during stabilisation and reperfusion also rendering larger infarct size and lower RPP compared to 6-week non-DM rats. The finding indicates that a similar pattern, with decreased metabolic flexibility, is seen with ageing[10]. The alterations associated with the duration of T2DM have been more extensively described in our earlier work[19].

The cardioprotective phenotype at onset diabetes has not only been demonstrated in animal models of T2DM[10], but also in models of type 1 diabetes using streptozotocin[30–33]. The underlying mechanisms may share similarities with cardioprotection by IPC, where major metabolic pathways are modulated[34]. A unifying pattern in animals cardioprotected by onset of diabetes and IPC seems to be that glucose oxidation rate at onset of reperfusion is low, and increases more rapidly after 3–5 minutes than in rats not achieving cardioprotection. The observation is consistent with a metabolic shutdown during ischemia and very early reperfusion followed by a gradual wake-up of metabolism during subsequent reperfusion[15].

Our study confirms that onset of diabetes yields endogenous protection against myocardial IR injury[10,30] and that diabetic hearts at late stage suffer increased IR-induced myocardial injury[19,23,35]. Increased IR-induced myocardial injury in late stage T2DM adds to the multiple factors that enhance morbidity and mortality after myocardial infarction in long duration of T2DM. In a translational approach, our present findings support the clinical experience that conditioning administered as remote IPC yields cardioprotection also in diabetes patients[12,13]. Overall, the beneficial effect seems to translate into a long-term beneficial clinical effect[36]. Whether the long-term beneficial reaches the same extent in diabetes patients as in patients without diabetes mellitus remains unknown.

We studied interstitial succinate concentrations because a recent study has demonstrated that succinate may act as a key mediator of IR injury[20]. While interstitial succinate concentrations was significantly decreased by IPC prior to ischemia, we also found that interstitial succinate concentration increases significantly at the end of ischemia after IPC with a subsequent rapid decrease during reperfusion, such that interstitial succinate levels were lower with than without IPC after 30 minutes of reperfusion consistent with the findings by Andrienko et al.[37] We observed a similar but less pronounced pattern with cardioprotection in diabetic animals and most evident in 12-week rats. The findings are in accordance with the findings by Sakamoto et al., who almost 20 years ago demonstrated that perfusion of hearts with succinate protected them from IR injury[38]. Moreover, not only treatment of hearts with fumarate[39,40] but also genetic knockdown of fumarate hydratase that causes fumarate accumulation[41] and increased succinate levels in the heart, are cardioprotective. A potential mechanism may be a decreased citric acid cycle (CAC) activity due to decreased consumption of reduced equivalents by the respiratory chain enzymes during early reperfusion. When induced in healthy hearts by respiratory chain enzymes inhibitors, the mechanism induces cardioprotection[42,43], and a reduced CAC flux[44] supporting this assumption. Our findings are not in accordance with a more recent finding that ischemic succinate accumulation represents a metabolic signature of ischemia and is responsible for mitochondrial ROS production during reperfusion[20]. Although ischemic succinate accumulation was present during ischemia, it was more modest in our model and the accumulation was amplified by IPC. Our findings yield support to the notion that significant superoxide production in the mitochondrial matrix, driven by succinate-fuelled reverse electron transport at mitochondrial complex I, occurs in the early phase of reperfusion[45].

We studied the metabolic changes by measuring glucose oxidation and interstitial metabolic concentrations. It is a limitation that we did not have the possibility to measure respiratory chain enzymes and western blotting of ischemia regulators.

We induced IPC by two cycles of 5 min ischemia + 5 min reperfusion prior to index ischemia. Choosing a remote IPC model might have enhanced the clinical applicability of our study. However, the IPC protocol is known as the strongest cardioprotective preconditioning stimulus and a very robust model, and was therefore chosen in this proof-of-concept study. Although the induction of intermittent myocardial ischemia by this artificial intervention may differ from short-lasting ischemic episodes by preinfarction angina, known to elicit naturally occurring IPC, some of the underlying mechanisms may vary. Hence, we cannot exclude that the efficacy of naturally occurring IPC may be attenuated in diabetic individuals and consequently modify outcome. However, our results support that diabetic individual may benefit from conditioning strategies during IR injury.

Despite inherent differences in sensitivity to ischemia reperfusion injury, the cardioprotective effect of IPC was preserved in our animal model of pre-, early- and late -stage T2DM and associated with adaptations in glucose oxidation capacity. These findings are in accordance with the clinical experience that diabetic patients suffering from AMI can benefit from the potential cardioprotective effect of conditioning.

## Supporting information

S1 Table Animals characteristics divided in groups ± succinate analysis performed on animal. (PDF)
