# Supplementary material for: Influence of diabetes mellitus duration on the efficacy of ischemic preconditioning in a Zucker diabetic fatty rat model
Source: PLoS One. 2018 Feb 23;13(2):e0192981. doi: 10.1371/journal.pone.0192981 (PMC5825060; doi:10.1371/journal.pone.0192981)
Supplement: S1 Table — (PDF) [file pone.0192981.s001.pdf]

Animal characteristics. Animals divided in groups ± succinate analysis performed on animal.

	**6-weeks-old rats**			
	non-DM		ZDF	
	all analysis *(n=12)*	no succinate analysis *(n=7)*	all analysis *(n=16)*	no succinate analysis *(n=2)*
Bodyweight (g)	151.2 ± 24.4	169 ± 19.8 ns	196.2 ± 16.7	202.5 ± 3.5 ns
Heartweight (mg)	651 ± 115	780 ± 243 ns	725 ± 177	773 ± 42 ns
HW/BW ratio	4.3 ± 0.4	4.6 ± 1.2 ns	3.7 ± 0.7	3.8 ± 0.1 ns
B-glucose (mmol/L)	4.9 ± 0.5	4.9 ± 0.8 ns	6.4 ± 1.4	6.5 ± 1.1 ns
P-total cholesterol (mmol/L)	2.2 ± 0.5	1.8 ± 0.6 ns	2.4 ± 0.9	1.9 ± 1.2 ns
P-triglyceride (mmol/L)	0.5 ± 0.2	0.4 ± 0.1 ns	1.9 ± 0.8	2.1 ± 0.8 ns

	**12-weeks-old rats**			
	non-DM		ZDF	
	all analysis *(n=14)*	no succinate analysis *(n=5)*	all analysis *(n=16)*	no succinate analysis *(n=4)*
Bodyweight (g)	306.2 ± 13.4	318.8 ± 11 ns	359.8 ± 21.3 ns	365.5 ± 13.8 ns
Heartweight (mg)	1061 ± 109	1106 ± 127 ns	1085 ± 69 ns	1073 ± 50 ns
HW/BW ratio	3.5 ± 0.4	3.5 ± 0.5 ns	3.0 ± 0.2	2.9 ± 0.2 ns
B-glucose (mmol/L)	5.0 ± 0.4	4.7 ± 0.3 ns	18.4 ± 6.4 ns	13 ± 4.3 ns
P-total cholesterol (mmol/L)	1.8 ± 0.2	1.4 ± 0.2 ns	3.8 ± 0.6 ns	3.3 ± 0.8 ns
P-triglyceride (mmol/L)	0.5 ± 0.07	0.4 ± 0.04 ns	6.5 ± 2.0 ns	5.3 ± 2.4 ns

	**24-weeks-old rats**			
	non-DM		ZDF	
	all analysis *(n=17)*	no succinate analysis *(n=2)*	all analysis *(n=10)*	no succinate analysis *(n=8)*
Bodyweight (g)	429.1 ± 20.8 ns	429 ± 18.4 ns	383.4 ± 30.1 ns	423.7 ± 30.9 ns
Heartweight (mg)	1431 ± 341 ns	1325 ± 40 ns	1245 ± 136 ns	1414 ± 537 ns
HW/BW ratio	3.3 ± 0.7	3.1 ± 0.04 ns	3.3 ± 0.3	3.5 ± 1.5 ns
B-glucose (mmol/L)	5.2 ± 0.3 ns	5.8 ± 0.2 ns	17.6 ± 3.1 ns	22.4 ± 4.2 ns
P-total cholesterol (mmol/L)	2.5 ± 1.1 ns	2.2 ± 0.2 ns	7.1 ± 0.9 ns	6.4 ± 1.9 ns
P-triglyceride (mmol/L)	0.7 ± 0.2 ns	0.6 ± 0.01 ns	7.0 ± 2.4 ns	8.2 ± 3.9 ns

Mean ± SD

ns: not significant compared to animal-matched all analysis
